# Supplementary material for: Multi-omics profiling of primary small cell carcinoma of the esophagus reveals RB1 disruption and additional molecular subtypes
Source: Nat Commun. 2021 Jun 18;12:3785. doi: 10.1038/s41467-021-24043-6 (PMC8213753; doi:10.1038/s41467-021-24043-6)
Supplement: Supplementary file 1 — Supplementary Information [file 41467_2021_24043_MOESM1_ESM.pdf]

Supplementary Information

Multi-omics profiling of primary small cell carcinoma of the esophagus reveals *RB1* disruption and additional molecular subtypes

Li *et al.*

Content

Supplementary Figure 1 Mutational burden and spectrum compared to other cancers

Supplementary Figure 2 Somatic mutations of *TP53* and *RB1* validated by RNA-seq

Supplementary Figure 3 *RB1* disruption by multiple mechanisms

Supplementary Figure 4 The transcriptomic landscape of PSCCE is similar to SCLC but different from ESCC or EAC

Supplementary Figure 5 PSCCE subtyping is associated with *MYC* amplification and similar to SCLC subtyping

Supplementary Figure 6 *POU2F3* and *YAP1* expressions are relatively low in 38 PSCCEs with RNA-seq

Supplementary Figure 7 Tumor microenvironment of PSCCE

Supplementary Table 1 Antibodies used in this study

Supplementary Table 2 PCR primers used in this study

Supplementary References

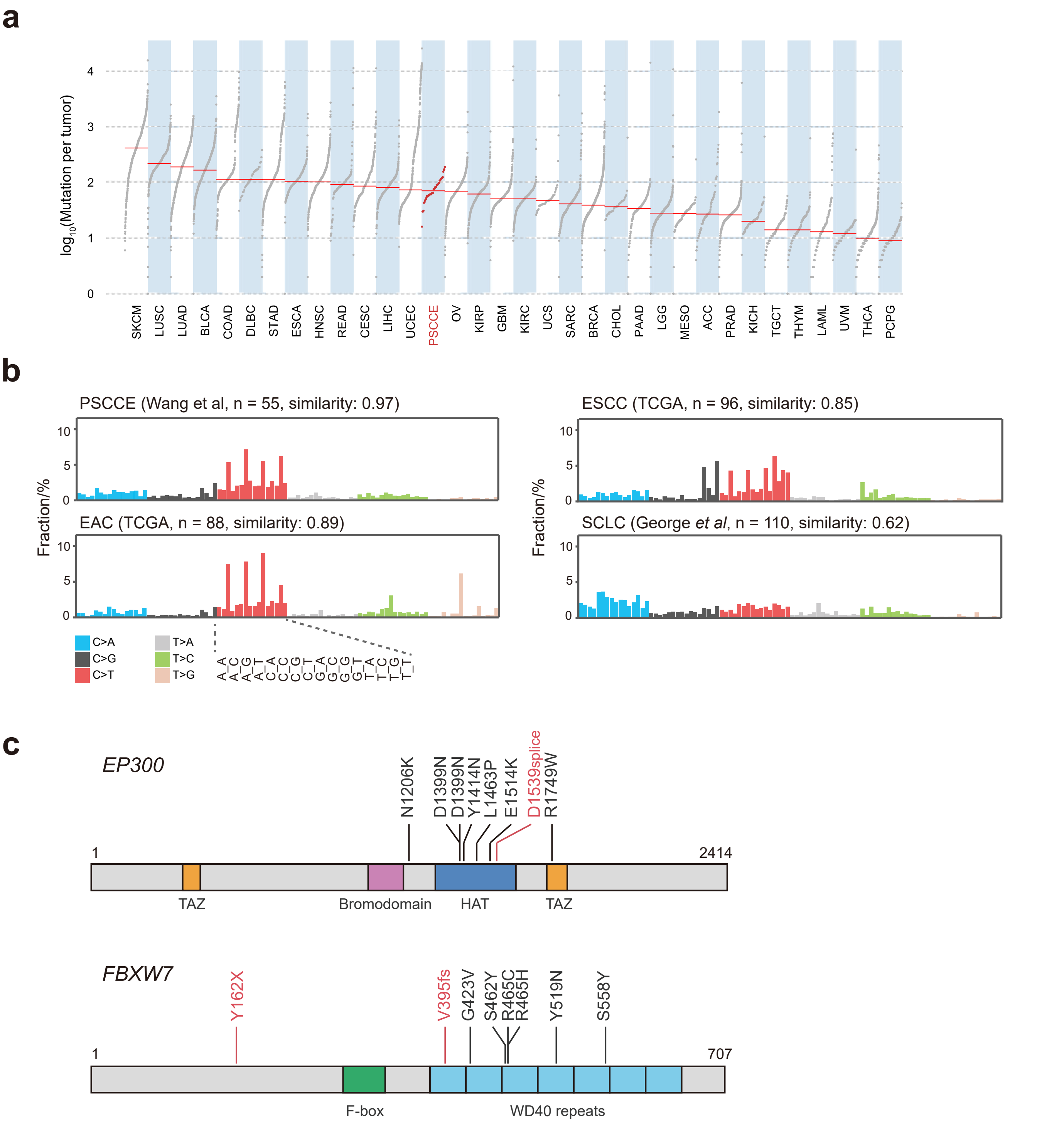

Supplementary Figure 1 Mutational burden and spectrum compared to other cancers. **(a)** Mutational burden of PSCCE (n = 46, the present study) compared to cancers sequence by The Cancer Genome Atlas (TCGA) project. Only nonsynonymous mutations are included. Medians are plotted as horizontal red line. **(b)** Mutation spectrums of PSCCE by Wang F *et al.*[1], EAC (TCGA, ref.[2]), ESCC (TCGA, ref.[2]) and SCLC by George J *et al.*[3]. Numbers of sequenced tumors and cosine similarities to the present study are shown. **(c)** Mutation in *EP300* and *FBXW7* identified in the combined cohort (n=101). Truncating mutations are colored in red.

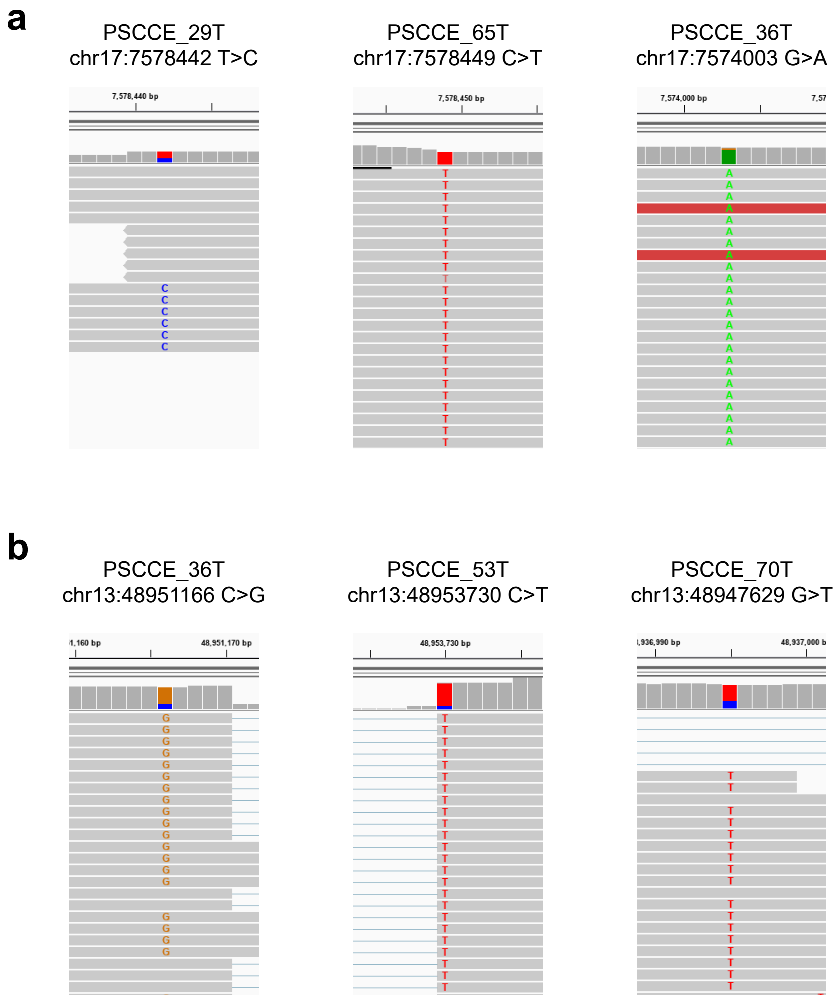

Supplementary Figure 2 Somatic mutations of **(a)** *TP53* and **(b)** *RB1* validated by RNA-seq. For loci that suffered PCR amplification failure, expression of somatic mutations in mRNA was checked by Integrated Genomic Viewer[4] (IGV). Sample names and somatic mutations detected by WES are shown above each IGV snapshot. Grey bars in IGV snapshots represent RNA-seq reads. Somatic mutations in RNA-seq reads are highlighted in colors.

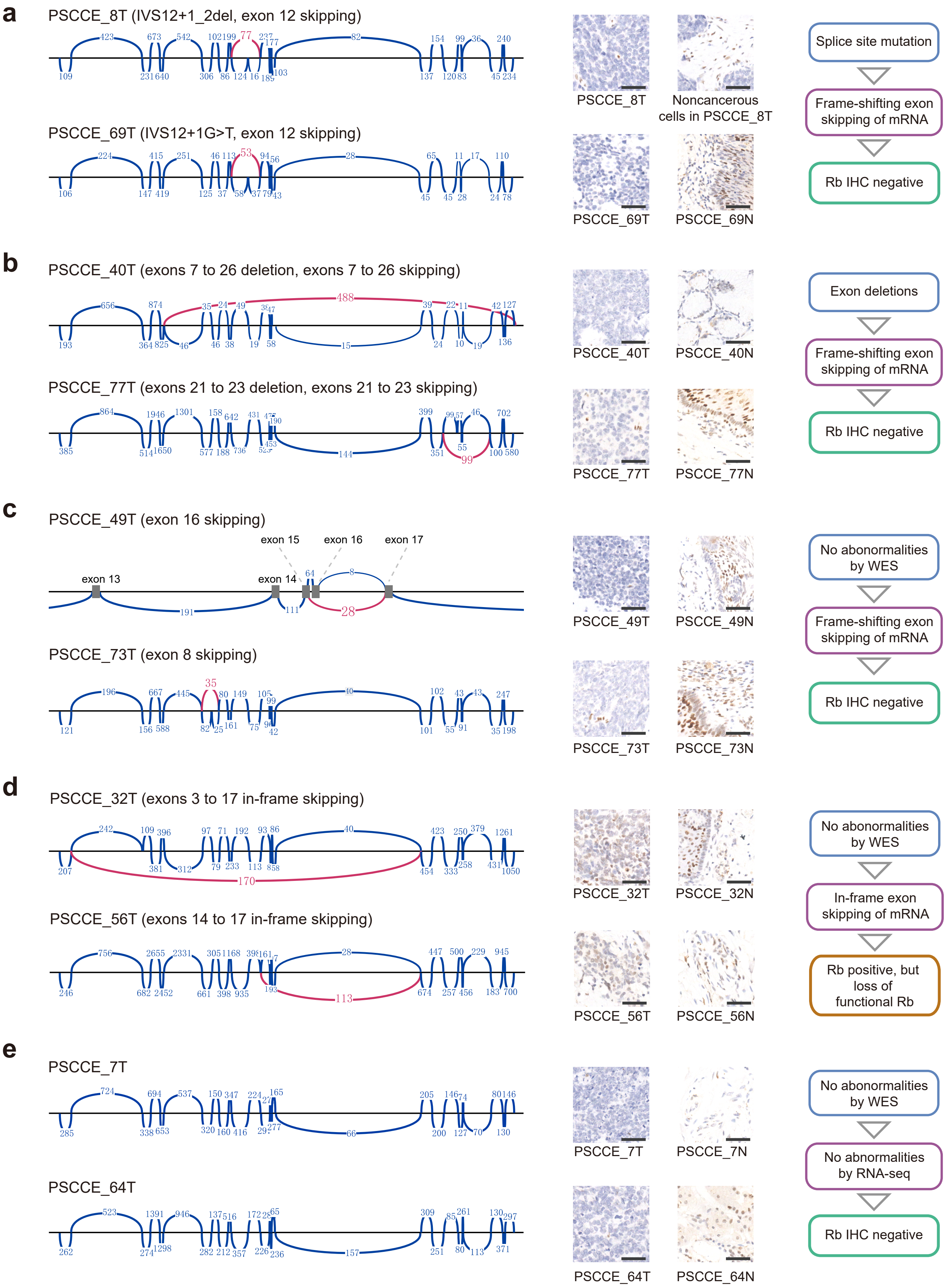

Supplementary Figure 3 *RB1* disruption by multiple mechanisms. In each panel, sashimi plots (left), Rb IHCs of tumor and matched normal tissue (middle) and schematic summary of *RB1* disruption events (right) are shown. **(a)** Splice-site mutation resulted in frame-shifting exon skippings and abolished Rb expression; **(b)** Exon deletions resulted in frame-shifting exon skippings and abolished Rb expression; **(c)** Frame-shifting exon skippings of *RB1* mRNA in tumors with no abnormalities by WES and negative Rb staining; **(d)** In-frame exon skippings of *RB1* mRNA in tumors with no abnormalities by WES and positive Rb staining; **(e)** In some tumors, no abnormalities were detectedby WES or RNA-seq while Rb IHC was negative. Curves in sashimi plot represent reads spanning exon junctions. Numbers of reads are denoted. Abnormal exon junctions are plotted in red. Scale bar: 50 μm.

a

Upregulated	DNA conformation change, DNA packaging<br>cell cycle phase transition, chromatin silencing<br>forebrain development, neuron fate commitment
Downregulated	muscle contraction, angiogenesis<br>cell-substrate adhesion, cell junction assembly<br>inflammatory response, leukocyte activation

b

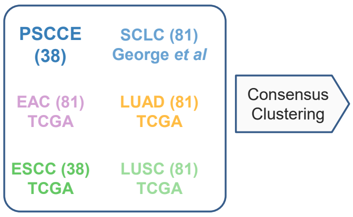

	Group NE	Group Squamous	Group Adeno
PSCCE	38	0	0
EAC	0	0	37
ESCC	0	38	0
SCLC	80	0	1
LUAD	0	2	79
LUSC	0	77	3

c

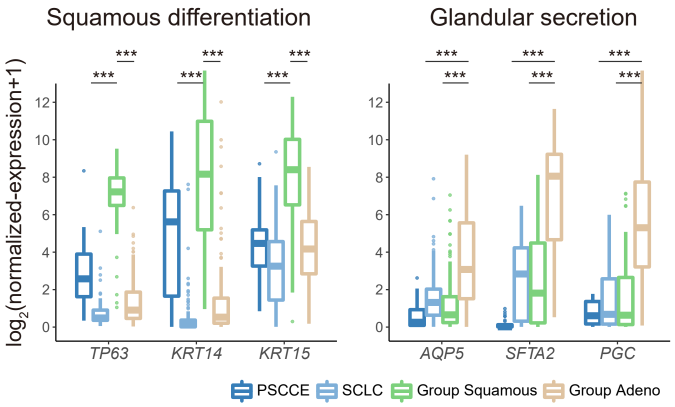

d

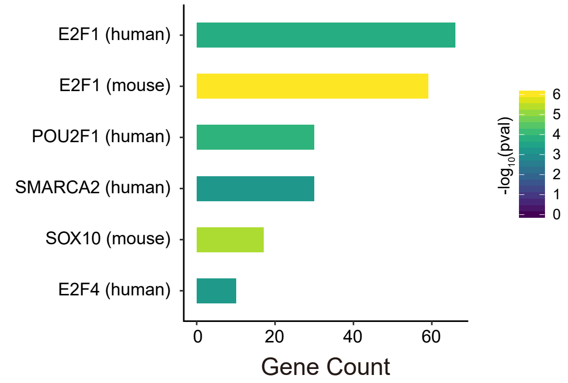

e

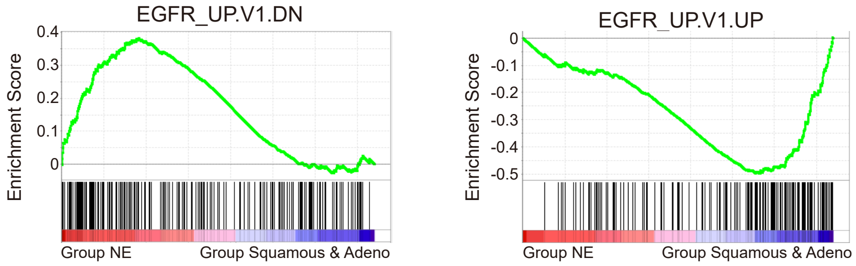

Description	Genes downregulated by EGFR overexpression	Genes upregulated by EGFR overexpression
Enrichment score	0.38	-0.50
P	0.0021	0.0009
q	0.038	0.023

Supplementary Figure 4 The transcriptomic landscape of PSCCE is similar to SCLC but different from ESCC or EAC. **(a)** Up- and downregulated biological processes enriched for differentially expressed genes of PSCCE. **(b)** Schematic summary of consensus clustering and resultant groups. **(c)** Squamous cell differentiation and glandular differentiation associated genes were specially expressed in Group Squamous and Group Adeno, respectively. ***: $P < 0.001$, by Wilcoxon rank-sum test. **(d)** Group NE signature genes were enriched for *E2F* targets. Enrichment analysis of transcriptional factors was performed on Enrichr[5] (https://maayanlab.cloud/Enrichr/). **(e)** Genes downregulated by hyperactive *EGFR* signaling pathway was preferentially expressed in Group NE while those upregulated were preferentially expressed in Group Squamous & Adeno.

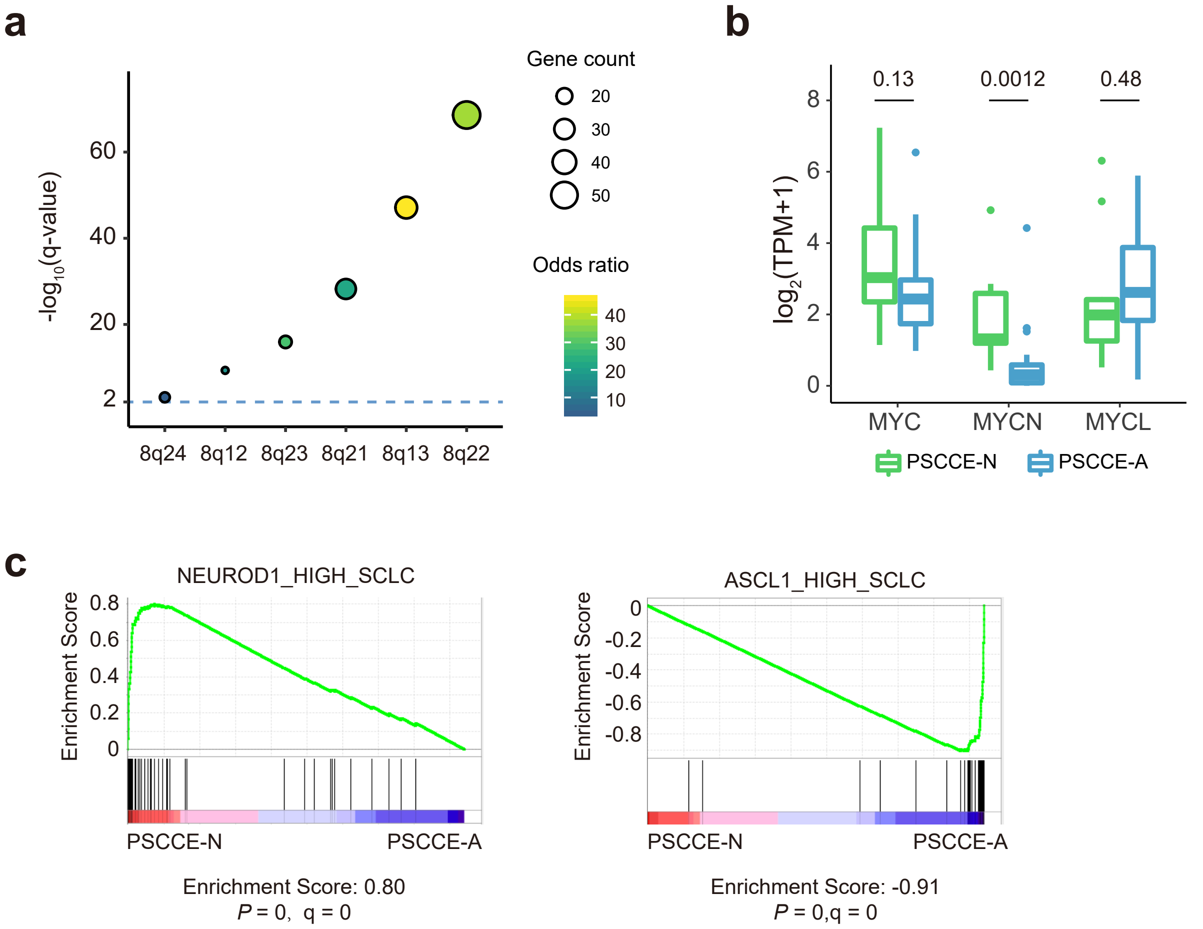

Supplementary Figure 5 PSCCE subtyping is associated with *MYC* amplification and similar to SCLC subtyping. **(a)** PSCCE-N subtype associated amplified genes were significantly enriched on chromosome 8q. Circle size represents number of genes enriched in each locus**.** Enrichment odds ratios compared to background are plotted as color gradient according to the color legend. Genomic location enrichment analysis was performed on Enrichr[5] (https://maayanlab.cloud/Enrichr/). **(b)** Comparison of *MYC* family genes expressions between two subtypes. *P* values were determined using Wilcoxon rank-sum test and shown on the plot. **(c)** Gene signatures of SCLC-N and SCLC-A tumors[6] were specially expressed in PSCCE-N and PSCCE-A tumors, respectively.

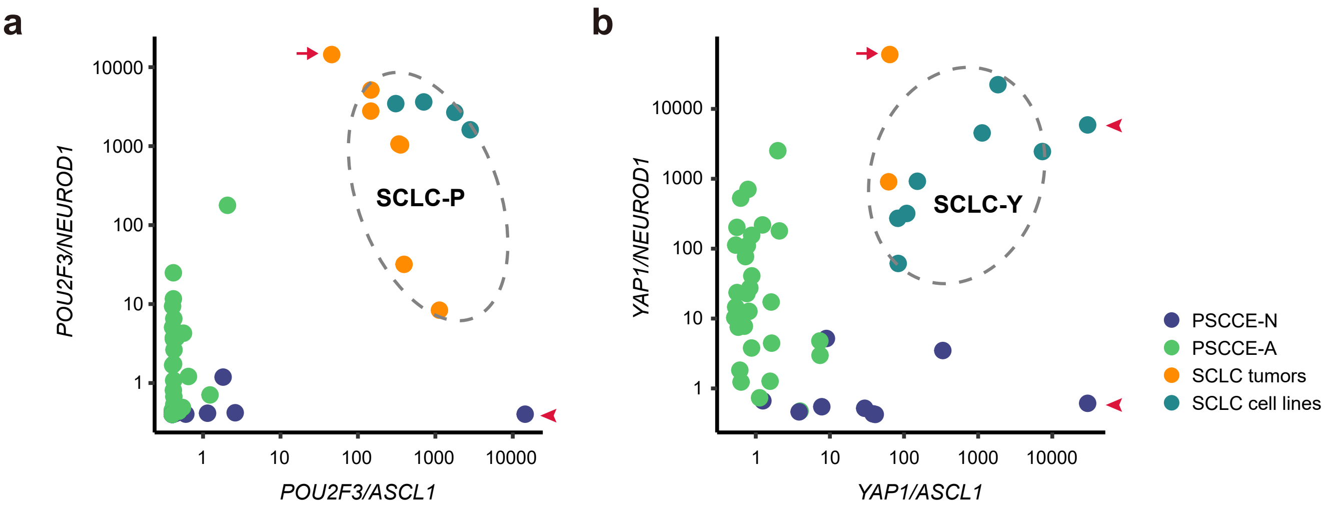

Supplementary Figure 6 *POU2F3* and *YAP1* expressions are relatively low in 38 PSCCEs with RNA-seq. **(a)** *POU2F3/NEUROD1* ratios are plotted against *POU2F3/ASCL1* ratios. PSCCEs are compared with reported SCLC-P tumors and cell lines. SCLC-P samples (grey dashed oval) showed both high *POU2F3/ASCL1* ratio and high *POU2F3/NEUROD1* ratio. PSCCEs had either high *ASCL1* or high *NEUROD1* thus located proximal to axes. The red arrowhead denotes one sample with infinite *POU2F3/ASCL1* ratio. The red arrow denotes one sample with infinite *POU2F3/NEUROD1* ratio. **(b)** *YAP1/NEUROD1* ratios are plotted against *YAP1/ASCL1* ratios. PSCCEs are compared with reported SCLC-Y tumors and cell lines. SCLC-Y samples (grey dashed oval) showed both high *YAP1/ASCL1* ratio and high *YAP1/NEUROD1* ratio. PSCCEs had either high *ASCL1* or high *NEUROD1* thus located proximal to axes. Red arrowheads denote samples with infinite *YAP1/ASCL1* ratio. The red arrow denotes one sample with infinite *POU2F3/NEUROD1* ratio. Gene expression values of SCLC cell lines were downloaded from Cancer Cell Line Encyclopedia[7] (CCLE, https://data.broadinstitute.org/ccle/CCLE_RNAseq_rsem_genes_tpm_20180929.txt.gz). Gene expression values of SCLC tumors were obtained from Supplementary Table 10 of George J *et al*.[3]. Names of SCLC-P and SCLC-Y samples were obtained from previous publications[8-10].

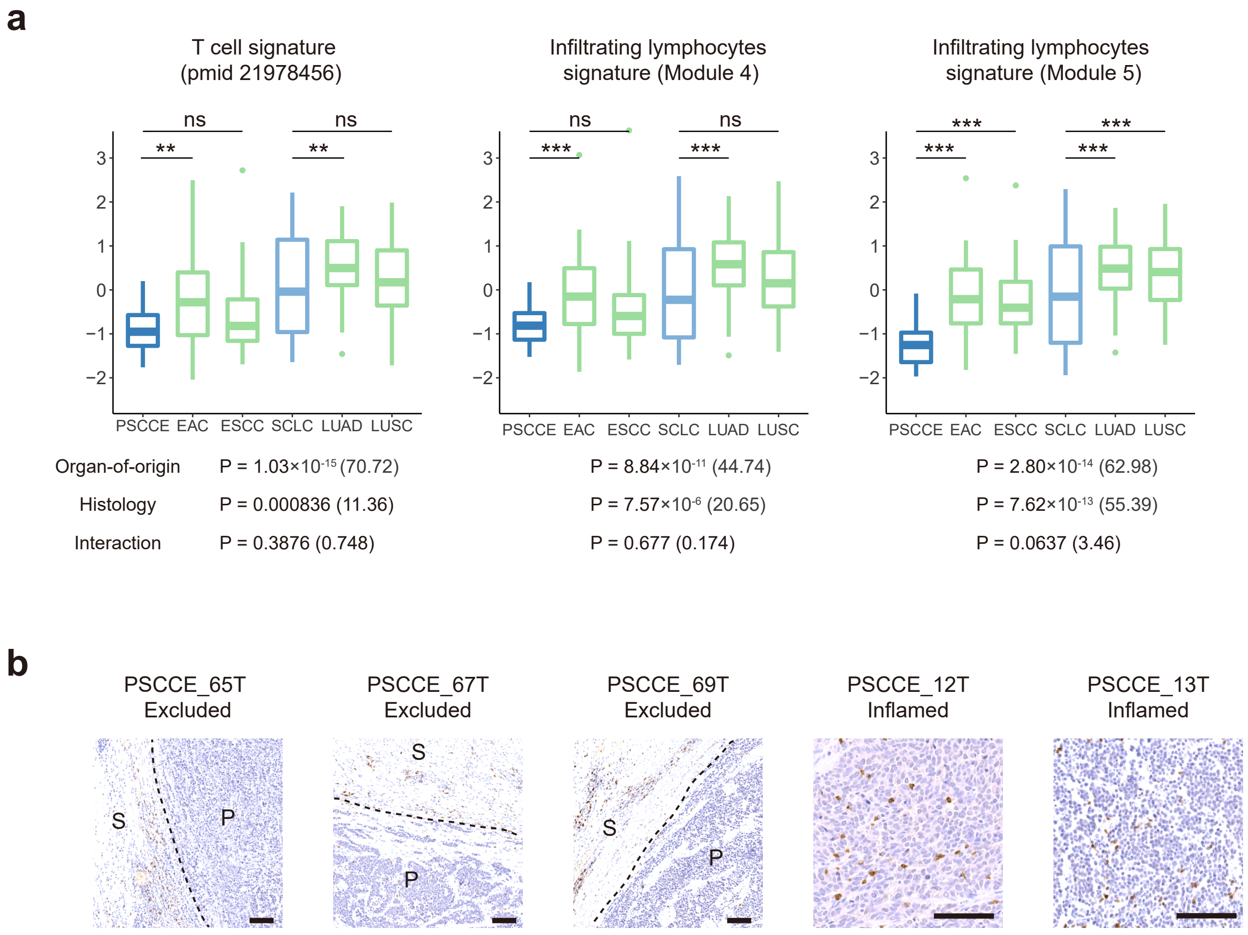

Supplementary Figure 7 Tumor microenvironment of PSCCE. **(a)** ssGSEA score of immune signatures. ** $P < 0.01$, *** $P < 0.001$, ns: not significant; all by Wilcoxon rank-sum test. Degree of freedom: organ-of-origin: 1; histology: 1. *F* values are shown in brackets following *P* values. **(b)** Representative fields of PSCCE CD8A IHC. In immune excluded tumors, $CD8^+$ T cells aggregated in surrounding stroma (S) near the invasive margin (dashed line) but failed to infiltrate into tumor parenchyma (P). In two inflamed tumors, $CD8^+$ T cells were in direct contact with tumor cells. Scale bar: 100 μm.

Supplementary Table 1 Antibodies used in this study

#	Target	Manufacturer	Cat No.	Dilution
1	*RB1*	Cell Signaling Technology	#9309	1/1000
2	*ASCL1*	abcam	ab74065	1/1000
3	*NEUROD1*	Sigma Aldrich	WH0004760M1	1/500
4	*CD8A*	Cell Signaling Technology	#85336S	1/100

Supplementary Table 2 PCR primers used in this study

#	Target	Forward	Reverse	Comment
1	*IFNG*	GCAGCCAACCTAAGCAAGAT	TCACCTGACACATTCAAGTTCT	qPCR validation of SCNV
2	*AQP5*	GGAAGAGCGGAAGAAGACCAT	CTCCTCCTCCTCAGCCAAGA	
3	*ACACA*	CAGCAGGTGAGTAGGAAGGT	CAGGCACAAATACAAGGCATAAG	
4	*ACLY*	GCACCGAAGACCAACATCC	GCAACATCCTAACGCCCTAC	
5	*MYC*	CTGCGACGAGGAGGAGAACT	CCGAAGGGAGAAGGGTGTGA	
6	*TERT*	CGTGGTTTCTGTGTGGTGTC	GGAGTAGAGGAAGTGCTTGGT	
7	*SOX4*	AACCAACAATGCCGAGAACAC	CGATCTGCGACCACACCAT	
8	PSCCE_10T/N	TGTGACCTTCAGCCAGAGTG	GTAAGATGCCAGACCTGTGAGT	PCR capture of RB1 exon deletion breakpoints
9	PSCCE_10T/N	TTATAGGCTCCAGATGAGTTAGG	AGGCTGAGGTTGCTTGTG	
10	PSCCE_13T/N	CGTTGCGGTGAATTATCCTTA	ATCCTCGGTTCTCTGTTCTTAT	
11	PSCCE_12T/N	AGCAGGTAGAGGAATCGTCAAT	GCAGAGCAGAGGATGGAGAG	
12	PSCCE_14T/N	TTATCTTCCACATCCAACCACAAG	TCACATTCTTCCTCCAGCAACT	
13	PSCCE_16T/N	GAGGTGGTGATGGTGATGCTA	CTGAAGAGTGTGGTGTGAGAAC	
14	PSCCE_8T/N	AGGCGAGGTCAGAACAGGA	CGGTAATACAAGCGAACTCCAA	RT-PCR capture of abnormal exon junctions in RB1 mRNA
15	PSCCE_10T/N	GCAGGTGTTATTCTAGGAGGAG	CAGACAGAAGGCGTTCACAA	
16	PSCCE_10T/N	AGATACCAGATCATGTCAGAGA	GGAAGATCCTTGTATGCTGTTA	
17	PSCCE_12T/N	AAGCAGAAGGCAACTTGACAA	GCATGAAGACCGAGTTATAGAATAC	
18	PSCCE_13T/N	CCTCTCGTCAGGCTTGAGTT	GTTGGTCCTTCTCGGTCCTT	
19	PSCCE_14T/N	CTACCTTGTCACCAATACCTCAC	GATCCTCATTTCTCTTCCTTGTTTG	
20	PSCCE_16T/N	CCTCTCGTCAGGCTTGAGTT	GTTGGTCCTTCTCGGTCCTT	

Supplementary References

1. Wang F*, et al.* The genomic landscape of small cell carcinoma of the esophagus. *Cell Res* **28**, 771-774 (2018).
2. Cancer Genome Atlas Research N*, et al.* Integrated genomic characterization of oesophageal carcinoma. *Nature* **541**, 169-175 (2017).
3. George J*, et al.* Comprehensive genomic profiles of small cell lung cancer. *Nature* **524**, 47-53 (2015).
4. Robinson JT*, et al.* Integrative genomics viewer. *Nature Biotechnology* **29**, 24-26 (2011).
5. Chen EY*, et al.* Enrichr: interactive and collaborative HTML5 gene list enrichment analysis tool. *BMC Bioinformatics* **14**, 128 (2013).
6. Borromeo MD*, et al.* ASCL1 and NEUROD1 reveal heterogeneity in pulmonary neuroendocrine tumors and regulate distinct genetic programs. *Cell Rep* **16**, 1259-1272 (2016).
7. Ghandi M*, et al.* Next-generation characterization of the Cancer Cell Line Encyclopedia. *Nature* **569**, 503-508 (2019).
8. Rudin CM*, et al.* Molecular subtypes of small cell lung cancer: a synthesis of human and mouse model data. *Nat Rev Cancer* **19**, 289-297 (2019).
9. Huang YH*, et al.* POU2F3 is a master regulator of a tuft cell-like variant of small cell lung cancer. *Genes Dev* **32**, 915-928 (2018).
10. McColl K*, et al.* Reciprocal expression of INSM1 and YAP1 defines subgroups in small cell lung cancer. *Oncotarget* **8**, 73745-73756 (2017).
