# Supplementary material for: Multi-omics profiling of primary small cell carcinoma of the esophagus reveals RB1 disruption and additional molecular subtypes
Source: Nat Commun. 2021 Jun 18;12:3785. doi: 10.1038/s41467-021-24043-6 (PMC8213753; doi:10.1038/s41467-021-24043-6)
Supplement: Supplementary file 3 — Description of Additional Supplementary Files [file 41467_2021_24043_MOESM3_ESM.pdf]

Description of Additional Supplementary Files

File name: Supplementary Data 1
Description: Supplementary Data 1 Overview of patients and samples

File name: Supplementary Data 2
Description: Supplementary Data 2 Somatic mutations detected by WES

File name: Supplementary Data 3
Description: Supplementary Data 3 Significant SCNV peaks identified by GISTIC2.0

File name: Supplementary Data 4
Description: Supplementary Data 4 qPCR validation of SCNVs

File name: Supplementary Data 5
Description: Supplementary Data 5 Significantly mutated genes and genes with mutation cluster in the present study (n=46) and in the combined cohort (n=101)

File name: Supplementary Data 6
Description: Supplementary Data 6 Sanger validation of somatic mutations in TP53, NOTCH1 and RB1

File name: Supplementary Data 7
Description: Supplementary Data 7 Deletions affecting RB1

File name: Supplementary Data 8
Description: Supplementary Data 8 Summary of RB1 mRNA splicing abnormalities observed in PSCCEs

File name: Supplementary Data 9
Description: Supplementary Data 9 Samples and resultant groups in unsupervised clustering

File name: Supplementary Data 10
Description: Supplementary Data 10 Signature genes of three groups

File name: Supplementary Data 11
Description: Supplementary Data 11 Subtype of each PSCCE

File name: Supplementary Data 12

Description: Supplementary Data 12 Signature genes of each subtype

File name: Supplementary Data 13

Description: Supplementary Data 13 Comparison of clinical features between two subtypes

File name: Supplementary Data 14

Description: Supplementary Data 14 Immune cell signatures for ssGSEA

File name: Supplementary Data 15

Description: Supplementary Data 15 Immune phenotype evaluation by CD8A IHC
